# Organ preservation in rectal cancer following clinical complete response after short-course radiotherapy-based total neoadjuvant therapy

**DOI:** 10.1016/j.ctro.2026.101216

**Published:** 2026-06-11

**Authors:** Carlos G. Morales, Erik Manriquez-Alegria, Valentina Duran, Maite Gonzalez, Gonzalo Carvajal, Pamela Briones, Felipe Mena, Martin Quintana, Richard Castillo, Angelo Fulle, Manuel Cabreras, Rodrigo Kusanovich, Nicole Caire, Javier Retamales, Patricia García, Diego Muñoz-Salazar, Felipe F. Quezada-Díaz

**Affiliations:** a Colorectal Surgery Unit, Complejo Asistencial Dr. Sótero del Río, Santiago, Chile; b Escuela de Medicina, Pontificia Universidad Católica de Chile, Santiago, Chile; c Institute of Science and Innovation in Medicine, Faculty of Medicine, Clínica Alemana–Universidad del Desarrollo, Santiago, Chile; d Center for Cancer Prevention and Control (FONDAP-CECAN), Santiago, Chile; e Medical Oncology Unit, Complejo Asistencial Doctor Sótero del Río, Santiago, Chile; f Department of Pathology, School of Medicine, Pontificia Universidad Católica de Chile, Santiago, Chile

**Keywords:** Rectal cancer, Short-course radiotherapy, Total neoadjuvant therapy, Watch-and-wait, Organ preservation, Hypofractionation

## Abstract

**Background and purpose:**

Organ preservation after total neoadjuvant therapy (TNT) is feasible in patients with locally advanced rectal cancer (LARC) who achieve a clinical complete response (cCR). However, most watch-and-wait (WW) evidence derives from long-course chemoradiotherapy (LCCRT), with limited data regarding short-course radiotherapy (SCRT)-based TNT. We evaluated response-adapted organ preservation following SCRT-based TNT in a public, resource-limited healthcare setting.

**Materials and methods:**

This retrospective cohort included consecutive patients with stage II–III LARC (cT3-T4 and/or N+) with palpable tumors ≤8 cm from the anal verge treated between 2020 and 2023. All patients received SCRT (25 Gy in five fractions) followed by consolidation chemotherapy (FOLFOX or CAPOX). Tumor response was assessed 8–12 weeks after TNT using digital rectal examination, pelvic MRI, flexible sigmoidoscopy and CEA levels. Patients achieving a cCR were managed with a structured WW protocol; others underwent total mesorectal excision (TME). Time-to-event outcomes were estimated descriptively.

**Results:**

Among 51 evaluable patients, 19 (37.3%) achieved cCR and 18 (35.3%) were managed with WW. After a median follow-up of 43.2 months (IQR 34.1–51.5), 3 WW patients (16.7%) developed local regrowth, all successfully salvaged with R0 resection. The estimated 24-month local regrowth-free survival was 82.2% (95% CI 65.8–100%). One WW patient died from systemic progression after salvage surgery. In the surgical group, 4 patients (12.1%) achieved a pathological complete response (pCR). Overall, 13 of 18 WW patients (72.2%) maintained sustained cCR without any oncologic event during follow-up.

**Conclusions:**

Hypofractionated SCRT integrated into a TNT strategy enabled response-adapted organ preservation with acceptable local regrowth-free survival in selected patients with LARC. This strategy was deliverable within a public healthcare system and achieved acceptable mid-term oncologic control. Prospective validation with longer follow-up is warranted.

## Highlights

• SCRT-based total neoadjuvant therapy achieved meaningful clinical complete response rates in LARC.
• A response-adapted watch-and-wait strategy enabled organ preservation in carefully selected patients.
• Local regrowth was infrequent and successfully managed with salvage surgery.
• Oncologic outcomes in patients achieving cCR were consistent with acceptable disease control.

## 1 Introduction

The management of locally advanced rectal cancer (LARC) has evolved significantly with the incorporation of total neoadjuvant therapy (TNT), which has improved both clinical and pathological complete response (pCR) rates [1]. Consequently, surgery—once the cornerstone of treatment—has assumed a secondary role in selected patients, making organ-preserving strategies a feasible option for a carefully selected subset of patients achieving a clinical complete response (cCR). Among these strategies, the watch-and-wait (WW) approach has become an accepted nonoperative alternative for patients achieving a cCR after TNT [2], [3]. This approach benefits patients by sparing them from the surgical morbidity and long-term sequelae associated with total mesorectal excision (TME) [4], [5], [6], [7], while maintaining oncologic outcomes comparable to surgery in selected cohorts [1], [8].

TNT is now widely adopted for LARC; however, the optimal radiotherapy backbone within TNT remains debated. While long-course chemoradiotherapy (LCCRT) has traditionally served as the standard platform [9], [10], short-course radiotherapy (SCRT; 25 Gy in 5 fractions) delivers hypofractionated pelvic irradiation over a condensed treatment schedule and has gained increasing interest within TNT protocols [11], [12]. When combined with consolidation chemotherapy, SCRT-based TNT has demonstrated favorable tumor regression and pCR rates, reduced distant metastases, and high treatment compliance [13].

Originally pioneered by Habr-Gama and colleagues in the early 2000s [6], [7], the WW approach has subsequently been validated by multiple international cohorts and registries [3], [14], [15], [16], and was more recently established as a feasible goal within prospective TNT protocols by the OPRA trial [17] and its long-term follow-up [18]. However, most WW evidence remains based on LCCRT, and data supporting SCRT-based TNT as a platform for response-adapted organ preservation remain limited and heterogeneous [15], [16]. In addition, emerging reports also suggest a potential risk of local regrowth following SCRT-based TNT, underscoring the need for further evaluation [19], [20].

Prolonged radiotherapy schedules and advanced delivery techniques may be less feasible in resource-constrained settings, where access and infrastructure limitations influence treatment selection. SCRT represents a potentially efficient and scalable alternative in such contexts. The primary aim of implementing this SCRT-based TNT protocol was to ensure systemic control and optimize resource utilization in a public healthcare setting with constrained radiotherapy capacity. However, a predefined secondary objective was to evaluate the feasibility and safety of a response-adapted organ preservation strategy in all patients achieving a cCR. We hypothesized that SCRT-based TNT allows a subset of patients to achieve and sustain a cCR under a WW approach, without evidence of compromised oncologic control, particularly in contexts where LCCRT is less feasible.

## 2 Methods and materials

### 2.1 Study design and patient selection

This retrospective single-center cohort study included all consecutive adult patients (≥18 years) with biopsy-proven rectal adenocarcinoma treated between January 1, 2020, and December 31, 2023, at a public tertiary hospital in “Anonymized for Review”. The full initial cohort (*n* = 62) was used for safety and feasibility analyses, while a subset (*n* = 51) who completed the protocol and underwent definitive management constituted the evaluable cohort for efficacy outcomes. Patients were identified using the institutional electronic medical record system. All cases were initially evaluated and followed by a single colorectal surgeon with dedicated training and expertise in WW protocols.

Eligible patients had clinical stage II–III disease (cT3–T4 and/or any N+), tumors located within 8 cm from the anal verge and clinically palpable on digital rectal examination (DRE), and an Eastern Cooperative Oncology Group (ECOG) performance status of 0–1.

Exclusion criteria included prior initiation of neoadjuvant therapy before institutional evaluation, T4 tumors requiring multivisceral resection, need for diverting stoma prior to TNT. To comprehensively assess treatment feasibility, safety was evaluated in the entire initial cohort who initiated treatment (*n* = 62). However, patients with failure to complete the planned treatment, loss to follow-up, or absence of definitive management were excluded from the primary efficacy analysis, resulting in a final evaluable cohort of 51 patients.

Data on demographics, tumor characteristics, treatment details, and outcome data were obtained from institutional databases and verified by chart review when necessary. The study was approved by the institutional ethics committee. Written informed consent was obtained from patients enrolled in the WW protocol.

### 2.2 Diagnostic workup

Baseline staging included complete colonoscopy with DRE, high-resolution pelvic MRI, contrast-enhanced computed tomography (CT) of the chest, abdomen, and pelvis, and serum carcinoembryonic antigen (CEA) measurement.

### 2.3 Simulation and treatment planning

Patients underwent CT simulation in the supine position using a flat tabletop with knee and foot supports for reproducibility. A non-contrast planning CT scan (3-mm slice thickness) was acquired from the L1 vertebral level to 5 cm below the perineum. Bladder filling was not mandated due to institutional resource limitations. Treatment planning was performed using the Monaco™ treatment planning system (Elekta AB, Stockholm, Sweden).

### 2.4 Target volume delineation

Target volumes were defined according to ICRU 50 and 62 recommendations. The gross tumor volume (GTV) included the primary tumor and suspicious mesorectal lymph nodes identified on baseline MRI. The clinical target volume (CTV) encompassed the mesorectum, the presacral nodal region (S1–S3), and bilateral internal iliac nodal regions, including the obturator and medial internal iliac stations. External iliac nodal chains were included only in selected cases with suspected anterior organ invasion, at the discretion of the treating radiation oncologist.

The planning target volume (PTV) was generated by applying a 1.0 cm expansion to the CTV and 0.7 cm to the GTV to account for setup uncertainty and organ motion. All contours were reviewed and approved by the attending radiation oncologist [21], [22].

### 2.5 Dose prescription and treatment delivery

All patients received SCRT, consisting of 25 Gy in five consecutive daily fractions of 5 Gy. The hypofractionated schedule corresponds to a biologically effective dose (BED10) of 37.5 Gy assuming an α/β ratio of 10 Gy for rectal adenocarcinoma. No simultaneous integrated or sequential boost was delivered to enlarged lateral pelvic lymph nodes; all target volumes received the prescribed dose of 25 Gy in 5 fractions.

Treatment was delivered using an Elekta Compact linear accelerator with 6-MV photon beams and a three-dimensional conformal radiotherapy (3D-CRT) technique. A four-field box arrangement (anteroposterior, posteroanterior, and opposed lateral fields) with a field-in-field approach was used to optimize dose homogeneity within the PTV.

Although intensity-modulated radiotherapy (IMRT) was not routinely available during the study period, the 3D-CRT approach provided adequate target coverage for short-course pelvic irradiation with acceptable organ-at-risk (OAR) sparing.

### 2.6 Organs at risk and quality assurance

OARs, including small bowel (bowel bag), bladder, femoral heads, and anal canal (for distal tumors), were contoured according to RTOG pelvic guidelines [23]. Planning objectives included: small bowel V25 Gy <10%, bladder V25 Gy <50%, femoral heads V25 Gy <5%, and anal canal maximum dose <27.5 Gy. Deviations due to tumor proximity were documented and approved by the treating radiation oncologist.

Machine output and beam quality were verified according to institutional quality assurance protocols following AAPM TG-51 recommendations [24]. No adaptive planning was performed.

### 2.7 Consolidation chemotherapy

Consolidation chemotherapy was preferably started within 11–18 days after completion of SCRT, but no later than 4 weeks. Chemotherapy regimens included either nine cycles of FOLFOX6 (oxaliplatin 85 mg/m^2^ intravenously on day 1, leucovorin [folinic acid] 400 mg/m^2^ intravenously on day 1, followed by bolus fluorouracil 400 mg/m^2^ on day 1 and fluorouracil 2400 mg/m^2^ in continuous infusion in 46 h, with a chemotherapy-free interval between days 3–14) or six cycles of CAPOX (capecitabine 1000 mg/m^2^ orally twice daily on days 1–14, oxaliplatin 130 mg/m^2^ intravenously on day 1).

Regimen selection was determined by a multidisciplinary oncologic committee based on patient characteristics and institutional availability. Prior to each chemotherapy cycle, patients underwent clinical evaluation, complete blood count, comprehensive metabolic panel, and toxicity assessment in accordance with institutional protocols. Formal endoscopic and radiological response assessment was deferred until 8–12 weeks after completion of the entire TNT regimen.

### 2.8 Response assessment

Tumor response was evaluated 8–12 weeks after completion of TNT using a multimodal protocol integrating DRE, flexible sigmoidoscopy, high-resolution pelvic MRI, and CEA levels. Rectal endoscopic ultrasound (REUS) was not routinely utilized, as it is often confounded by radiation-induced fibrosis. All assessments were reviewed in a multidisciplinary committee including a colorectal surgeon and an experienced abdominal radiologist.

MRI restaging included T2-weighted sequences and diffusion-weighted imaging. Tumor regression was graded according to the MRI tumor regression grade (mrTRG) classification.

cCR required absence of palpable tumor on DRE, endoscopic evidence of a flat scar without ulceration or nodularity, according to criteria adapted from the Memorial Sloan Kettering Cancer Center response schema [25], including favorable MRI findings (mrTRG 1–2 without suspicious diffusion restriction), absence of suspicious nodal disease, and stable or decreasing CEA levels.

Near-complete response (nCR) cases were reassessed after 6–8 additional weeks. Patients whose response deepened to a cCR were enrolled in the WW protocol, while those with persistent clinical, endoscopic, or radiologic evidence of residual tumor were classified as iCR and referred for TME.

### 2.9 Treatment decision

Patients achieving cCR were offered a WW strategy. Those with iCR proceeded to TME (low anterior resection or abdominoperineal resection according to tumor characteristics). Treatment decisions were made through shared decision-making within a multidisciplinary committee.

### 2.10 Follow-up and surveillance

Patients achieving a cCR were offered a structured WW surveillance protocol, adapted from international recommendations and adjusted to local resource availability. Surveillance was intensified during the first 24 months, when the risk of local regrowth is highest.

Evaluations included DRE, CEA measurement, and flexible sigmoidoscopy every four months; pelvic MRI every six months; and annual CT imaging. Surveillance frequency was subsequently reduced after two years. Between years 3 and 5, clinical evaluation with DRE, CEA measurement, and flexible sigmoidoscopy was performed every six months, pelvic MRI annually, and CT imaging annually [26]. Patients undergoing TME were followed according to NCCN guidelines [27].

### 2.11 Definitions and outcomes

Local regrowth was defined as tumor recurrence within the rectal wall after cCR. Sustained cCR was defined as absence of local regrowth during follow-up in patients with WW.

Disease-free survival (DFS) was defined as absence of local regrowth (WW group), local recurrence after TME, or distant metastases. Overall survival (OS) was defined as time from treatment completion to death from any cause.

Organ preservation was defined as avoidance of TME during follow-up, regardless of whether local excision was performed.

The primary outcome was local regrowth-free survival (LRFS) among patients managed with a WW strategy, defined as the time from WW enrollment to the date of biopsy-confirmed local regrowth or last follow-up. The median time to local regrowth was reported descriptively among patients who experienced this event. LRFS was estimated using the Kaplan–Meier method. Secondary outcomes included pCR among surgically treated patients, DFS and OS.

### 2.12 Statistics

Continuous variables were reported as medians with interquartile ranges (IQR). Categorical variables were analyzed using Fisher's exact test. Time-to-event outcomes, including local regrowth-free survival, DFS, and OS, were estimated using the Kaplan–Meier method and reported descriptively without formal comparative testing (e.g., log-rank test) due to the response-adapted design of the study. Deaths without prior local regrowth were treated as censored events; no such events occurred in the WW cohort, so this analytical choice did not affect LRFS estimates. Follow-up was calculated from the end of TNT for the WW cohort and from the date of surgery for the TME cohort, introducing an inherent offset of 20–28 weeks reflecting the protocol-specified restaging interval (8–12 weeks) and surgical waiting times in our public healthcare system (12–16 weeks). All statistical analyses were performed using RStudio (version 2023.12.0 + 369; Posit Software, PBC, Boston, MA, USA). This study was conducted and reported in accordance with the STROBE guidelines [28].

### 2.13 Data availability

Research data are stored in an institutional repository and will be shared upon request to the corresponding author.

## 3 Results

**Fig. 1:**
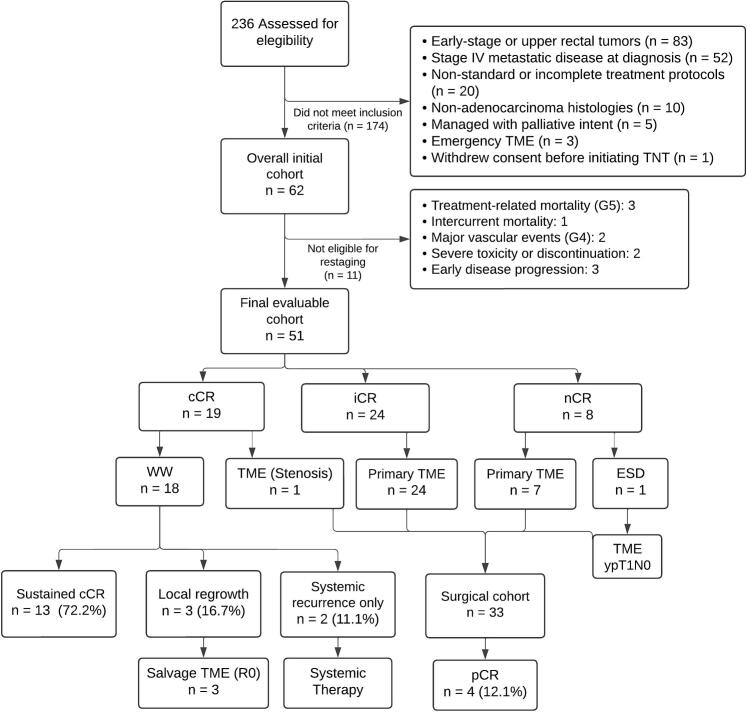
Flow diagram of patient selection and treatment pathways. Flowchart illustrating patient inclusion, exclusion, and final allocation to Watch-and-Wait (WW) or total mesorectal excision (TME) following SCRT-based TNT. Abbreviations: cCR = clinical complete response; nCR = near complete response; iCR = incomplete response; pCR = pathological complete response.

A total of 62 patients with LARC initiated the SCRT-TNT protocol. Patient demographics and baseline tumor characteristics for the entire cohort are summarized in Table 1. Of the initial cohort, 11 patients (17.7%) were excluded from the efficacy analysis due to clinical events: treatment-related mortality (*n* = 3), intercurrent mortality (*n* = 1), major vascular events (*n* = 2), severe toxicity precluding restaging (n = 2), and early disease progression (n = 3) **(**Table 2**)**. The final evaluable cohort consisted of 51 patients. The patient selection process and treatment pathways are summarized in Fig. 1**.** Among the 51 evaluable patients, 19 (37.3%) achieved a cCR at restaging; of these, 18 (35.3%) were enrolled in the WW protocol, while one patient with severe symptomatic post-radiation rectal stenosis precluding endoscopic surveillance underwent TME and was analyzed within the surgical cohort (final pathology: ypT0N0, R0). Of the remaining 32 patients with non-complete response, 24 had an iCR and 8 had a nCR. Among the nCR patients, one underwent endoscopic submucosal dissection (ESD) for a suspicious residual lesion, which confirmed ypT1 adenocarcinoma; this patient subsequently proceeded to radical TME (final pathology: ypT1N0, R0). The remaining 31 non-complete responders proceeded directly to primary TME, yielding a final surgical cohort of 33 patients.

**Table 1:** Baseline clinical and tumor characteristics.

Variable	Overall initial cohort** (*n* = 62) No. (%)	WW (*n* = 18) No. (%)	TME (*n* = 33) No. (%)
Age, y, median [IQR]	63 [53–70]	65 [53–68]	57 [50–70]
Sex			
Male	35 (57.4)	8 (44.4)	23 (69.7)
Female	26 (42.6)	10 (55.6)	10 (30.3)
BMI, median [IQR]	27.7 [24.6–30]	30.8 [26–32.4]	27 [24.6–28.5]
Tobacco use	26 (44.1)	7 (38.9)	16 (50)
ECOG performance status			
0	17 (29.3)	6 (33.3)	9 (28.1)
1	41 (70.7)	12 (66.7)	23 (71.9)
Serum CEA, ng/ml, median [IQR]	3.4 [2.2–6.4]	2.7 [1.7–6.5]	3.6 [2.5–6.4]
Tumor distance from anal verge on MRI, median [IQR], cm	6 [4–8]	5 [4–8]	7 [4–7.5]
Major tumor size, median [IQR], cm	5.8 [5–7]	5.3 [4.1–6]	6.5 [5.07–8]
Extramural venous invasion positive	22 (37.9)	3 (16.7)	16 (51.6)
MRF involvement ≤1 mm	25 (43.9)	6 (33.3)	14 (46.7)
Sphincter complex invasion	10 (16.4)	3 (16.7)	5 (15.2)
Mesorectal lymph nodes involvement	52 (89.7)	14 (77.8)	31 (93.9)
Enlarged lateral nodes	27 (50)	10 (55.6)	15 (48.4)
Clinical T stage			
T2	6 (10.3)	3 (16.7)	2 (6.1)
T3	39 (67.2)	15 (83.3)	20 (60.6)
T4	13 (22.4)	0 (0)	11 (33.3)
Clinical N stage			
N0 (negative)	4 (6.9)	3 (16.67)	1 (3.03)
N+ (positive)	54 (93.1)	15 (83.3)	32 (97)

Abbreviations: BMI, body mass index; CEA, carcinoembryonic antigen; ECOG, Eastern Cooperative Oncology Group; IQR, interquartile range; MRF, mesorectal fascia; TME, total mesorectal excision; WW, Watch-and-Wait. Percentages reflect available data; missing values (1–8 per variable) were not imputed. **Overall initial cohort: includes the 11 patients excluded from the final efficacy analysis (detailed in Table 2), in addition to the 18 WW and 33 TME patients shown in the adjacent columns.

**Table 2:** Clinical reasons for protocol discontinuation and exclusion from the evaluable cohort.

Category	Patients, n (%)	Specific Events or Clinical Rationale
Treatment-related mortality (Grade 5)	3 (27.3)	Pulmonary embolism (n = 2); neutropenic enterocolitis (n = 1)
Intercurrent mortality	1 (9.1)	Unrelated traumatic event
Major vascular events (Grade 4)	2 (18.2)	Ischemic stroke (n = 1); status epilepticus (n = 1)
Severe toxicity or discontinuation	2 (18.2)	Grade 4 febrile neutropenia (n = 1); deep vein thrombosis and grade 3 ileocolitis (n = 1)
Early disease progression	3 (27.3)	Tumor perforation (n = 1); systemic progression (n = 2)
Total	11 (100)

One excluded case merits explicit description. After SCRT and 5 cycles of FOLFOX (discontinued due to deep vein thrombosis and grade 3 actinic ileocolitis), this patient had an iCR at restaging and declined the recommended abdominoperineal resection to avoid a permanent stoma. After 21 months without protocol-defined management, the patient reconsidered and underwent R0 resection (ypT3N0), remaining free of disease. As this trajectory deviated from the response-adapted pathway, the patient was excluded from the efficacy cohort but accounted for in the intention-to-treat denominator (Table 2).

Among the initial cohort of 62 patients, the median age was 63 years (IQR 53–70), 26 patients (42.6%) were female, the median serum CEA was 3.4 ng/mL (IQR 2.2–6.4), and the median tumor distance from the anal verge was 6 cm (IQR 4–8). Detailed baseline clinical and radiological characteristics are presented in Table 1.

In the WW subgroup, baseline MRI demonstrated mesorectal nodal involvement in 14 cases (77.8%), a threatened circumferential resection margin in 6 cases (33.3%), and extramural vascular invasion in 3 cases (16.7%). In the evaluable cohort (*n* = 51), the most frequent adverse events during consolidation chemotherapy were peripheral neuropathy, neutropenia, fatigue, and gastrointestinal toxicity, consistent with the expected profile of FOLFOX6 and CAPOX regimens.

After a median follow-up of 43.2 months (IQR 34.1–51.5), three of the 18 WW patients (16.7%) developed local regrowth at a median of 14.8 months (IQR 9.1–22.8) from WW enrollment. All three underwent salvage TME with R0 margins (two LAR, one APR). Two patients remained disease-free at last follow-up, whereas one developed lung and brain metastases after salvage surgery and died 38 months after initial diagnosis. Two additional patients developed distant metastases without preceding local regrowth and remain alive on systemic therapy. Thirteen of the 18 WW patients (72.2%) maintained a sustained cCR without any oncologic event during follow-up. Overall, the WW cohort accrued one disease-related death during the observation period.

The cumulative incidence of local regrowth in the WW cohort reached a plateau of 16.7% at 24 months, with no further events observed during extended follow-up **(**Fig. 3**)**.

The estimated local regrowth-free survival was 88.5% (95% CI 74.8–100%) at 12 months and 82.2% (95% CI 65.8–100%) at both 24 and 36 months. At last follow-up, 15 patients (29.4% of the evaluable cohort) avoided TME during follow-up: 13 with sustained cCR without any oncologic event, and 2 with isolated distant metastases managed with systemic therapy.

In the surgical cohort (*n* = 33), one patient died in the perioperative period due to hemoperitoneum and anastomotic leakage. Final pathological staging demonstrated a pathological complete response (ypT0N0) in 4 patients (12.1%), ypT1 in 2 patients (6.1%), ypT2 in 6 patients (18.2%), ypT3 in 18 patients (54.5%), and ypT4 in 2 patients (6.1%); one specimen was non-evaluable for ypT. Mesorectal lymph node involvement (ypN+) was present in 13 patients (39.4%) **(**Table 3**).**

**Table 3:** Surgical and pathological findings in the TME group.

Variable	TME (n = 33) No. (%)
Resection type	
LAR*	24 (72.7)
APR*	9 (27.3)
Resection margins	
R0	31 (93.9)
R1	2 (6.1)
R2	0 (0)
Ryan Grade	
0	4 (12.1)
1	3 (9.1)
2	14 (42.4)
3	12 (36.4)
Pathological tumor classification (ypT)	
T0	4 (12.1)
T1	2 (6.1)
T2	6 (18.2)
T3	18 (54.5)
T4	2 (6.1)
Tx	1 (3)
Pathological nodal classification (ypN)	
N (−)	20 (60.6)
N (+)	13 (39.4)
Pathological Complete Response (pCR)	4 (12.1)

Outcomes following total mesorectal excision, including resection type, margin status, pathological tumor and nodal stage, and tumor regression grades. Abbreviations: LAR = low anterior resection; APR = abdominoperineal resection; pCR = pathological complete response; ypT/ypN = post-treatment pathological staging.

Positive circumferential and/or distal resection margins were observed in two patients (6.1%). The median follow-up for the surgical cohort was 28.4 months (IQR 13.6–38.7). During this period, 5 patients (15.2%) experienced local recurrence, and 11 patients (33.3%) developed distant metastases **(**Table 4**)**.

**Table 4:** Treatment parameters, clinical response, and oncological outcomes.

Variable	WW (*n* = 18) No. (%)	TME (n = 33) No. (%)
Consolidation Chemotherapy:		
FOLFOX6 x 9 cycles	16 (94.1)	29 (87.9)
CAPOX x 6 cycles	1 (5.9)	4 (12.1)
Clinical Response		
cCR	18 (100)	1 (3)
nCR	–	8 (24.2)
iCR	–	24 (72.7)
Median follow-up, months, [IQR]	43.2 [34.1–51.5]	28.4 [13.6–38.7]
24-month DFS, % (95% CI)	77.0 (49.7–90.7)	49.4 (31.0–65.5)
24-month OS, % (95% CI)	100	74.6 (55.6–86.4)
Locoregional failure and distant metastasis		
Local regrowth	3 (16.7)	–
Local recurrence	–	5 (15.2)
Distant metastasis	2 (11.1)	11 (33.3)
Death	1 (5.6)	11 (33.3)

Details of chemotherapy regimens, treatment responses, follow-up duration, survival outcomes, and recurrence events by treatment group. Abbreviations: cCR = clinical complete response; nCR = near complete response; iCR = incomplete response; DFS = disease-free survival; OS = overall survival; CI = confidence interval. Patients are categorized according to their first oncologic event during follow-up; therefore, one WW patient who developed distant metastases following local regrowth is counted under “Local regrowth” rather than “Distant metastasis.” Survival estimates derived from Kaplan-Meier method with 95% CIs computed using the log-log transformation.

**Fig. 2:**
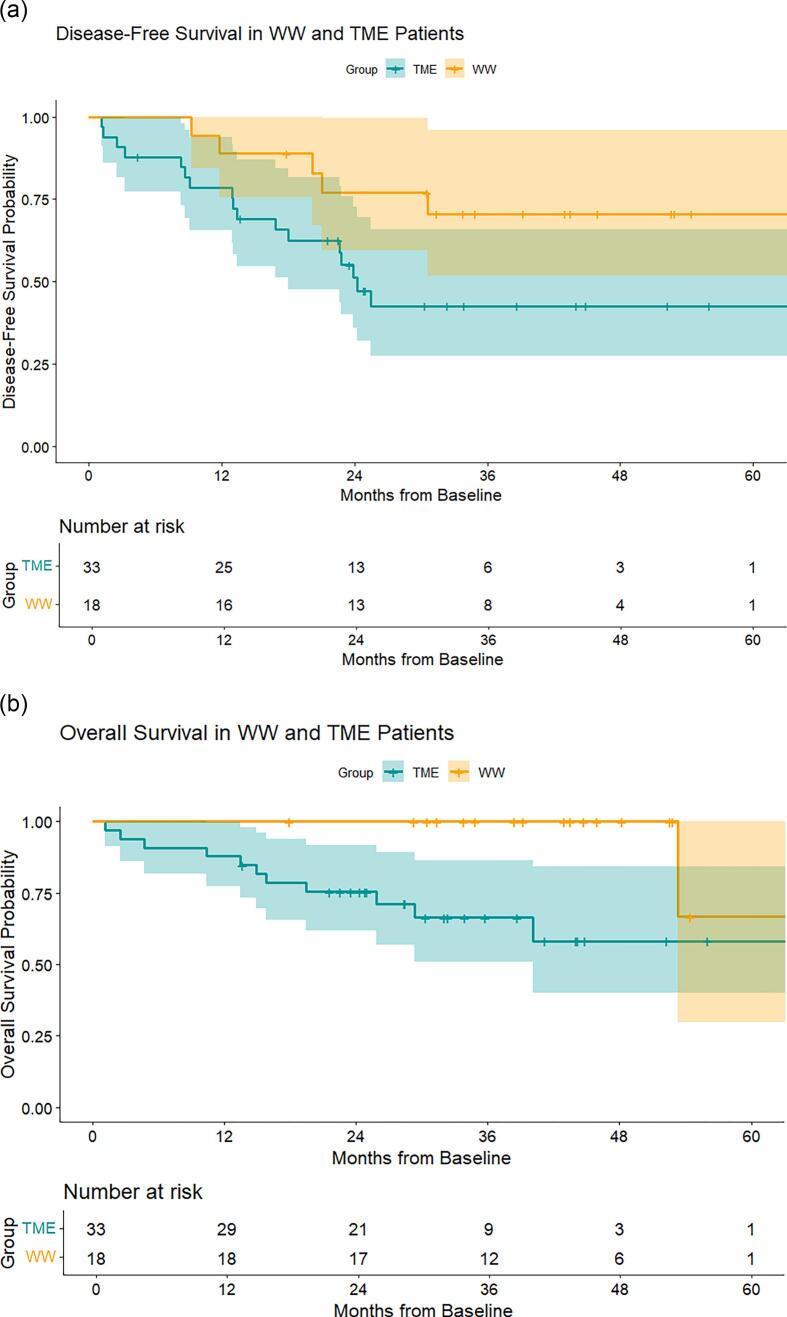
Disease-free survival and overall survival by treatment strategy. Kaplan–Meier estimates of (a) disease-free survival (DFS) and (b) overall survival (OS) for patients managed with Watch-and-Wait (WW) versus total mesorectal excision (TME). Shaded areas represent 95% confidence intervals; tick marks indicate censored observations.

Overall, 22 patients (43.1%) of the cohort achieved either clinical or pCR following SCRT-based TNT. At a median follow-up of 43.2 months for the WW group and 28.4 months for the surgical group, Kaplan-Meier estimates for DFS and OS are shown in Fig. 2a and b. The 24-month DFS was 77.0% (95% CI 49.7–90.7) in the WW group and 49.4% (95% CI 31.0–65.5) in the surgical group, while the 24-month OS was 100% in the WW group and 74.6% (95% CI 55.6–86.4) in the surgical group. Median DFS was not reached in the WW cohort and was 23.9 months in the surgical cohort; median OS was not reached in either group. The cumulative incidence of recurrence for both clinical trajectories is illustrated in Fig. 4. Survival outcomes are presented descriptively without formal comparative testing due to the response-adapted study design.

**Fig. 3:**
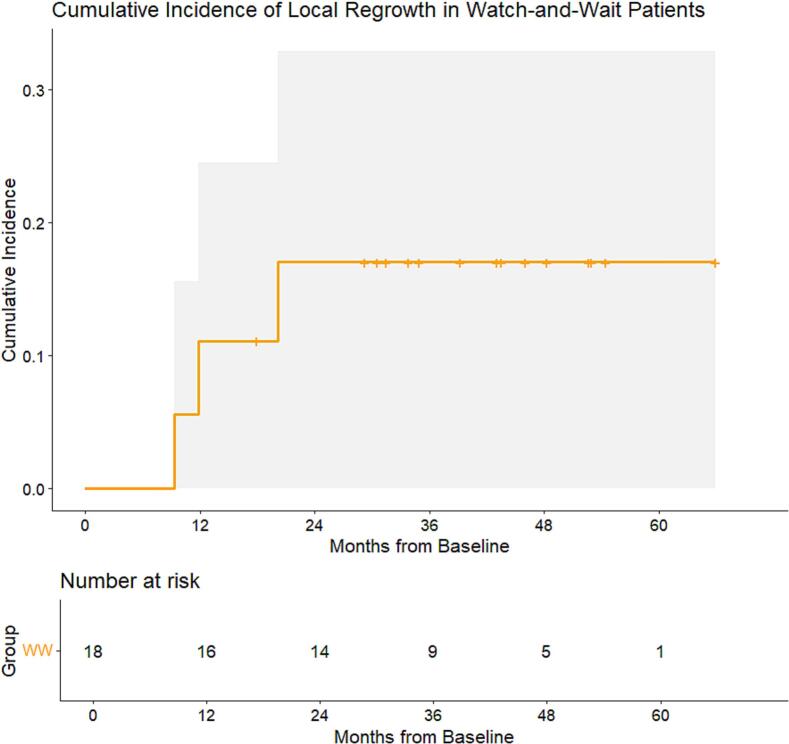
Cumulative incidence of local regrowth. Cumulative incidence of local regrowth in the Watch-and-Wait (WW) cohort (n = 18). Tick marks indicate censored observations.

**Fig. 4:**
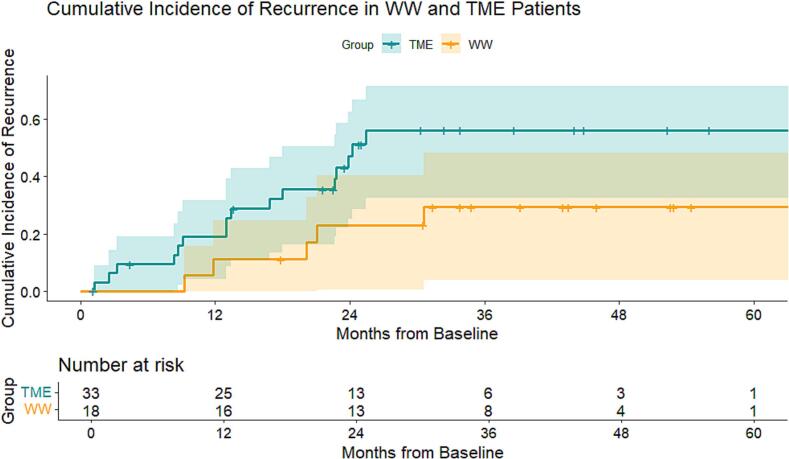
Cumulative incidence of recurrence by treatment strategy. Cumulative incidence of any recurrence (local or distant) stratified by Watch-and-Wait (WW) versus total mesorectal excision (TME). Shaded areas represent 95% confidence intervals; tick marks indicate censored observations.

## 4 Discussion

This retrospective cohort evaluates SCRT followed by consolidation chemotherapy as part of TNT, and its potential role in enabling response-adapted organ preservation in selected patients with LARC. In our cohort, 19 patients (37.3%) achieved a cCR; of these, 18 (35.3%) were managed with WW and 1 underwent TME because of post-radiation rectal stenosis (final pathology ypT0N0). Among patients with non-complete clinical response who proceeded to surgery, 3 additional pCR were identified on final pathology. Thus, a total of 22 patients (43.1%) achieved an overall complete response (cCR or pCR). Unlike the RAPIDO trial, which focused on distant metastasis control without a specific organ preservation aim, our protocol proactively integrated a WW pathway. Our higher complete response rate (43.1% vs. RAPIDO's 28%) likely reflects the inclusion of cCR alongside pCR (a more permissive composite endpoint), the exclusion of extreme high-risk cases (cT4b requiring multivisceral resection), and an intentionally delayed assessment (8–12 weeks) that may favor maximal tumor regression. These response rates are consistent with contemporary TNT trials [29], [30], and suggest that SCRT-based TNT can induce meaningful tumor regression, allowing organ preservation in a substantial proportion of patients even within a public, resource-constrained healthcare setting.

When contextualized against contemporary evidence on response-adapted organ preservation, our results align with published prospective data, although direct comparisons must be interpreted with caution. The OPRA trial (LCCRT-based TNT) reported a 5-year organ preservation rate of approximately 50%, with most local regrowth events occurring within the first two years after restaging [17], [18]. More directly comparable to our cohort, the recent retrospective analysis by Bercz et al. [20] compared LCCRT- and SCRT-based TNT regimens within the same institution and reported similar cCR rates (44.5% vs 43.4%) but a higher 2-year local regrowth rate among WW patients treated with SCRT (36%) compared with LCCRT (19%). Our observed cumulative incidence of local regrowth at 24 months (16.7%) is more favorable than the SCRT cohort reported by Bercz et al. and falls within the range described for LCCRT-based regimens. All local regrowths in our series were salvaged with R0 resection, consistent with both the OPRA trial and contemporary single-center series [3], [14], [31]. Whether the lower local regrowth rate observed in our cohort reflects favorable patient selection (exclusion of cT4b disease), the extended restaging interval (8–12 weeks), or other unmeasured factors warrants further evaluation. Particularly in resource-constrained public healthcare systems, the logistical advantage of reducing radiotherapy from 25 to 28 fractions to a 5-day schedule makes SCRT a clinically meaningful alternative that yields competitive complete response rates, a question that will be further evaluated in the ongoing ACO/ARO/AIO-18.1 trial (NCT04246684).

Earlier studies have specifically evaluated SCRT plus consolidation chemotherapy in the context of organ preservation. Jia et al. [32] reported a 47% cCR rate among 26 patients with stage II–III LARC treated with SCRT followed by mFOLFOX6 or CapOx, with most achieving WW-based organ preservation. Kim et al. [33], in a prospective trial of 19 patients, reported a 1-year cCR rate of 68% and identified involved circumferential resection margin as a predictor of lower cCR (40% vs. 93%). Together with our findings, these data support the feasibility of SCRT-based TNT for organ preservation, while highlighting that outcomes are influenced by baseline tumor burden, duration of consolidation chemotherapy, and the interval to response assessment.

From a radiation oncology perspective, SCRT delivers hypofractionated pelvic irradiation. Although SCRT delivers a lower overall BED to the tumor compared to conventional LCCRT, its higher dose per fraction (5 × 5 Gy) and very short overall treatment time facilitate the rapid integration of intensive systemic therapy without compromising local control. The use of larger fraction sizes may enhance direct tumor cell kill through increased DNA damage and reduced opportunity for sublethal repair compared with conventional LCCRT. When incorporated into TNT with delayed surgery or nonoperative management, this approach may permit ongoing tumor regression during consolidation chemotherapy, partially compensating for the absence of concurrent radiosensitization during irradiation. However, the use of hypofractionation raises theoretical concerns regarding increased late toxicity, including fibrosis and bowel dysfunction, due to higher dose per fraction effects on normal tissues [34]. Although late adverse events were not systematically evaluated in our cohort, prospective studies incorporating standardized toxicity and functional outcomes are required to fully characterize the long-term safety profile of SCRT-based TNT [19]. The use of a standardized hypofractionated pelvic protocol with predefined organ-at-risk constraints enhances reproducibility and may facilitate implementation across institutions with limited access to advanced radiotherapy techniques such as IMRT.

Beyond the radiobiological rationale, the optimal interval between radiotherapy completion and response assessment remains a relevant consideration. The timing of clinical evaluation may substantially influence cCR detection and, consequently, eligibility for WW. In this study, response assessment was performed 8–12 weeks after TNT completion, consistent with established practice. Nevertheless, emerging evidence suggests that extending this interval may increase the likelihood of identifying delayed complete responses, potentially enhancing organ preservation rates [35], [36]. Further investigation is needed to define the optimal assessment window within SCRT-based TNT protocols.

While disease-free and overall survival outcomes in the WW group were favorable, these findings must be interpreted cautiously. Patients achieving a cCR are inherently more likely to harbor biologically favorable tumors and to respond better to therapy, as previously reported [37]. Thus, the observed outcomes reflect effective disease control in a selected population rather than treatment superiority. Conversely, the surgical cohort demonstrated high rates of local recurrence and distant metastasis. These outcomes were associated with adverse baseline tumor characteristics — including greater proportions of cT4 disease, mesorectal fascia involvement, extramural vascular invasion, and residual ypT3–T4 disease — likely reflecting advanced tumor biology rather than limitations of the SCRT platform. An additional consideration is the prolonged interval between radiotherapy and definitive surgery (approximately 6–8 months including consolidation chemotherapy and restaging), which may theoretically allow repopulation of radioresistant clones in non-responders, potentially contributing to local progression or distant spread.

An important contribution of this study is the detailed description of treatment delivery, response assessment, and surveillance within a resource-constrained public healthcare setting. Surveillance intensity was adapted to balance international recommendations with local limitations in imaging availability and appointment capacity, while remaining sufficiently rigorous to allow early detection of local regrowth. This pragmatic approach may be informative for centers facing similar structural limitations.

Our findings contribute to the ongoing effort to refine patient selection and standardize surveillance for nonoperative management in rectal cancer. The ongoing NOM-ERA trial (NRG Oncology) is prospectively evaluating the safety and efficacy of watch-and-wait after TNT, including both CRT- and SCRT-based regimens, and is expected to provide high-level evidence to further define response-adapted strategies [38]. In parallel, a regional prospective multicenter phase II study in “Anonymized for Review” is currently assessing SCRT-based TNT followed by watch-and-wait in patients achieving cCR, incorporating patient-reported outcomes and translational biomarkers [39]. This trial is expected to generate region-specific prospective data supporting the feasibility and safety of organ preservation strategies within Latin American public healthcare systems.

Several limitations must be acknowledged. The retrospective, single-center design and modest sample size limit generalizability, and follow-up duration remains insufficient for definitive conclusions regarding long-term local control, late toxicity, and functional outcomes. The disparity in median follow-up between the WW (43.2 months) and surgical (28.4 months) cohorts reflects the distinct time zero used for each group (end of TNT vs. date of surgery), which introduced an inherent offset of 20–28 weeks, and the earlier oncologic events in the TME cohort that further shortened individual follow-up. In addition, the exclusion of one patient who declined recommended surgery after TNT and ultimately underwent delayed R0 resection at 21 months may introduce a minor selection bias when interpreting organ preservation outcomes, illustrating the relevance of patient preference and real-world management decisions in resource-constrained settings. We also acknowledge a significant inherent allocation bias: because group assignment was strictly response-adapted, the TME cohort predominantly comprises non-responders with potentially more aggressive tumor biology. Consequently, survival outcomes between the WW and TME groups are presented descriptively and cannot be formally compared. Radiotherapy was delivered using three-dimensional conformal techniques rather than IMRT, which may increase dose exposure to surrounding organs at risk. Acute and late toxicity were not systematically graded according to CTCAE criteria, and patient-reported functional outcomes were not collected. Future prospective studies should incorporate standardized toxicity reporting and quality-of-life assessment to better characterize the risk–benefit balance of SCRT-based TNT [40].

## 5 Conclusions

SCRT-based TNT represents a feasible radiotherapy platform for response-adapted organ preservation in selected patients with LARC treated in a resource-constrained public healthcare system. With careful patient selection and structured surveillance, a WW strategy achieved acceptable mid-term oncologic control. Integrating SCRT into TNT protocols may expand access to organ-preserving approaches, particularly in settings where LCCRT is not practical. Prospective studies with longer follow-up are warranted to further define patient selection, long-term safety, and optimal integration within TNT strategies

## Declaration of AI use

During the preparation of this work, the author(s) used Claude AI for grammar and spelling correction. After using this tool/service, the author(s) reviewed and edited the content as needed and take(s) full responsibility for the content of the published article.

## Funding

This research was funded by Agencia Nacional de Investigación y Desarrollo (ANID)/Fondo Nacional de Desarrollo Científico y Tecnológico (FONDECYT) Grant #11201291 (F.Q.D), and partially by ANID/FONDECYT Grant #1241269 (P.G.) and ANID/Fondo de Financiamiento de Centros de Investigación en Áreas Prioritarias (FONDAP) Grant #152220002 CECAN (F.Q.D & P.G.). The funder had no role in study design, data collection, data analysis, interpretation of results, or drafting of the manuscript.

## Declaration of competing interest

The authors declare that they have no known competing financial interests or personal relationships that could have appeared to influence the work reported in this paper.
