# Supplementary material for: Organ preservation in rectal cancer following clinical complete response after short-course radiotherapy-based total neoadjuvant therapy
Source: Clin Transl Radiat Oncol. 2026 Jun 11;60:101216. doi: 10.1016/j.ctro.2026.101216 (PMC13315657; doi:10.1016/j.ctro.2026.101216)
Supplement: Supplementary file 1 — Supplementary material [file mmc1.docx]

**STROBE Statement — Checklist of Items That Should Be Included in Reports of Cohort Studies**

*Organ Preservation in Rectal Cancer Following Clinical Complete Response After Short-Course Radiotherapy-Based Total Neoadjuvant Therapy*

| **Item No.** | **Recommendation** | **Page No.** |
| --- | --- | --- |
| **Title and Abstract** | | |
| 1a | Indicate the study's design with a commonly used term in the title or the abstract | 1 |
| 1b | Provide in the abstract an informative and balanced summary of what was done and what was found | 1–2 |
| **Introduction** | | |
| 2 | Explain the scientific background and rationale for the investigation being reported | 3–4 |
| 3 | State specific objectives, including any prespecified hypotheses | 4 |
| **Methods** | | |
| 4 | Present key elements of study design early in the paper | 4 |
| 5 | Describe the setting, locations, and relevant dates, including periods of recruitment, exposure, follow-up, and data collection | 4–5 |
| 6a | Give the eligibility criteria, and the sources and methods of selection of participants. Describe methods of follow-up | 5, 8–9 |
| 6b | For matched studies, give matching criteria and number of exposed and unexposed | N/A |
| 7 | Clearly define all outcomes, exposures, predictors, potential confounders, and effect modifiers. Give diagnostic criteria, if applicable | 9 |
| 8 | For each variable of interest, give sources of data and details of methods of assessment (measurement). Describe comparability of assessment methods if there is more than one group | 5, 7–8 |
| 9 | Describe any efforts to address potential sources of bias | 10, 16 |
| 10 | Explain how the study size was arrived at | 4–5 |
| 11 | Explain how quantitative variables were handled in the analyses. If applicable, describe which groupings were chosen and why | 10 |
| 12a | Describe all statistical methods, including those used to control for confounding | 10 |
| 12b | Describe any methods used to examine subgroups and interactions | 10 |
| 12c | Explain how missing data were addressed | Table 1 footnote |
| 12d | If applicable, explain how loss to follow-up was addressed | Response letter (0 lost) |
| 12e | Describe any sensitivity analyses | N/A |
| **Results** | | |
| 13a | Report numbers of individuals at each stage of study — eg numbers potentially eligible, examined for eligibility, confirmed eligible, included in the study, completing follow-up, and analysed | 10–11, Fig. 1 |
| 13b | Give reasons for non-participation at each stage | 10, Table 2 |
| 13c | Consider use of a flow diagram | Fig. 1 |
| 14a | Give characteristics of study participants (eg demographic, clinical, social) and information on exposures and potential confounders | 11, Table 1 |
| 14b | Indicate number of participants with missing data for each variable of interest | Table 1 footnote |
| 15 | Report numbers of outcome events or summary measures over time | 11–12, Tables 3–4 |
| 16a | Give unadjusted estimates and, if applicable, confounder-adjusted estimates and their precision (eg, 95% CI). Make clear which confounders were adjusted for and why | 12, Table 4 |
| 16b | Report category boundaries when continuous variables were categorized | N/A |
| 16c | If relevant, consider translating estimates of relative risk into absolute risk for a meaningful time period | N/A |
| 17 | Report other analyses done — eg analyses of subgroups and interactions, and sensitivity analyses | 11–12 |
| **Discussion** | | |
| 18 | Summarise key results with reference to study objectives | 13 |
| 19 | Discuss limitations of the study, taking into account sources of potential bias or imprecision. Discuss both direction and magnitude of any potential bias | 15–16 |
| 20 | Give a cautious overall interpretation of results considering objectives, limitations, multiplicity of analyses, results from similar studies, and other relevant evidence | 13–16 |
| 21 | Discuss the generalisability (external validity) of the study results | 15–16 |
| **Other Information** | | |
| 22 | Give the source of funding and the role of the funders for the present study and, if applicable, for the original study on which the present article is based | Funding section |

*N/A = not applicable. Page numbers refer to the revised manuscript.*
